# Development and Validation of a Novel Prognostic Nomogram for CD5-Positive Diffuse Large B-Cell Lymphoma: A Retrospective Multicenter Study in China

**DOI:** 10.3389/fonc.2021.754180

**Published:** 2021-11-03

**Authors:** Ziyuan Shen, Ling Wang, Bingpei Zhang, Tianci Li, Dashan Li, Chenlu He, Yuhao Xue, Ying Wang, Bingzong Li, Qinhua Liu, Hao Zhang, Weiying Gu, Fei Wang, Chunling Wang, Yuye Shi, Jingjing Ye, Taigang Zhu, Yuqing Miao, Shuiping Huang, Wei Sang

**Affiliations:** ^1^ Department of Epidemiology and Biostatistics, School of Public Health, Xuzhou Medical University, Xuzhou, China; ^2^ Department of Hematology, Taian Central Hospital, Taian, China; ^3^ Department of Hematology, Affiliated Hospital of Xuzhou Medical University, Xuzhou, China; ^4^ Department of Hematology, Huai’an First People’s Hospital, Huai’an, China; ^5^ Department of Personnel, Suqian First Hospital, Suqian, China; ^6^ Department of Hematology, The Second Affiliated Hospital of Soochow University, Suzhou, China; ^7^ Department of Hematology, The First Affiliated Hospital of Anhui Medical University, Hefei, China; ^8^ Department of Hematology, The Affiliated Hospital of Jining Medical University, Jining, China; ^9^ Department of Hematology, The First People’s Hospital of Changzhou, Changzhou, China; ^10^ Department of Hematology, Qilu Hospital of Shandong University, Jinan, China; ^11^ Department of Hematology, The General Hospital of Wanbei Coal-Electric Group, Suzhou, China; ^12^ Department of Hematology, Yancheng First People’s Hospital, Yancheng, China; ^13^ Center for Medical Statistics and Data Analysis, School of Public Health, Xuzhou Medical University, Xuzhou, China

**Keywords:** CD5-positive, DLBCL, nomogram, prognosis, validation

## Abstract

**Background:**

CD5-positive diffuse large B-cell lymphoma (CD5+ DLBCL) is a rare subtype of DLBCL with invasive clinical features and poor prognosis. Current clinical variables based on prognostic systems for DLBCL are inadequate to accurately stratify the prognosis of CD5+ DLBCL.

**Methods:**

A total of 195 CD5+ DLBCL patients were retrospectively recruited from nine centers in Huaihai Lymphoma Working Group. MaxStat analysis was used to identify optimal cutoff points for continuous variables; univariable and multivariable Cox analyses were used for variable selection; Kaplan–Meier curve was used to analyze the value of variables on prognosis; and C-index, Brier score, and decision curve analysis were measured for predicting model performance.

**Results:**

The derivation and validation cohorts consisted of 131 and 64 patients. Of the whole cohort, median age at diagnosis was 61 years, of whom 100 (51.28%) were males and the 5‐year overall survival rate was 42.1%. MYC, BCL-2, and the coexpression of MYC/BCL-2 could distinguish the survival of CD5+ DLBCL. Multivariable analysis showed that age, IPI, red blood cell count, neutrophil count, MYC expression, and hepatosplenomegaly were independent predictors, and the prognostic nomogram was developed. The C‐index of the nomogram was 0.809 in the derivation and 0.770 in the validation cohort. Decision curve analysis proved that compared with IPI, the specific nomogram showed a better identification in CD5+ DLBCL.

**Conclusion:**

The proposed nomogram provided a valuable tool for prognosis prediction in patients with CD5+ DLBCL.

## Introduction

Diffuse large B‐cell lymphoma (DLBCL) is a group of clinically invasive non‐Hodgkin’s lymphomas that are highly heterogeneous in terms of clinical manifestations, pathophysiological features, cell-of-origin (COO), and gene-based molecular stratification. Despite the current frontline regimen of rituximab-based immunotherapy that can cure many patients, there are still 40% of patients who experience relapse or remain refractory (1). The International Prognostic Index (IPI) has been the basis for predicting the survival of DLBCL, which could stratify patients into four risk groups (2). NCCN-IPI, an enhanced system with the capacity of discriminating low-risk and high-risk groups, is more powerful than IPI for predicting survival in the rituximab era (3). However, both of them are based on clinical factors, with the limitation in providing information on biological features (4). Additionally, due to the high heterogeneity of DLBCL, the identification of specific subtypes and the development of accurate prognostic models are badly needed for individualized treatment.

Elements of heterogeneity are associated with the prognosis of DLBCL. According to the gene expression profile-based classification of COO, the activated B-cell-like (ABC) subtype exhibits a worse prognosis than the germinal center B-cell-like (GCB) subtype (5). Patients of DLBCL with MYC rearrangement had a lower survival rate compared with those without MYC rearrangement (6). TP53 mutation in DLBCL has also been confirmed to be negative with prognosis (7–9). In addition, MYC/BCL-2 double expression was associated with poor outcomes in DLBCL patients (10–12). CD5-positive (CD5+) DLBCL was defined as an immunohistochemical subtype in the fourth edition of the World Health Organization (WHO) classification, which takes up approximately 5% to 10% of DLBCL (13, 14). CD5+ DLBCL always occurs in the elderly and the majority of patients belong to the ABC subtype, showing more invasive clinical course, central nervous system involvement (CNS involvement), and extranodal lymphadenopathy (15, 16). It is worth noting that CD5+ DLBCL patients do not benefit from rituximab-based immunochemotherapy and intensive regimens, and their 5-year survival rate is only 40% (17–19). Several studies have proven that clinicopathologic features were the prognosis of CD5+ DLBCL patients, while the majority of these studies have limitations of having small single-center samples (19–21). A precise prognostic stratification system for CD5+ DLBCL needs to be further explored for prognostic evaluation and individualized treatment.

Nomograms are commonly used to estimate the prognosis of patients, which can provide a statistical predictive model and generate an individual numerical probability by integrating diverse prognostic factors (22). The visual format of nomograms is a simpler, more sophisticated tool with numerous advantages and is readily understood by physicians and patients. It has been demonstrated in studies of many malignancies including breast cancer, gastric cancer, and lymphoma (23–25).

In this retrospective study, we retrieved 195 CD5+ DLBCL cases from the Huaihai Lymphoma Working Group (HHLWG) and analyzed the clinicopathological characteristics of CD5+ DLBCL, aiming to develop and validate a novel prognostic nomogram for individual prognosis evaluation.

## Materials and Methods

### Patient Cohort

Data from two centers of HHLWG in this study served as the derivation cohort. The two centers are 1) The Affiliated Hospital of Xuzhou Medical University and 2) Huai’an First People’s Hospital. Data from seven centers of HHLWG in this study served as the external validation cohort. The seven centers are 1) The Affiliated Hospital of Jining Medical University, 2) The First People’s Hospital of Changzhou, 3) Yancheng First People’s Hospital, 4) The First Affiliated Hospital of Anhui Medical University, 5) Qilu Hospital of Shandong University, 6) The Second Affiliated Hospital of Soochow University, and 7) The General Hospital of Wanbei Coal-Electric Group. Study approval was obtained from the independent ethics committees of each participating center in HHLWG and met the Helsinki Declaration. All patients were retrieved from the above centers between March 1, 2010, and January 19, 2021.

### Baseline Characteristics of Patients

The following variables were collected: age, gender, lactate dehydrogenase (LDH), platelet (PLT), lymphocyte count (LYC), albumin (Alb), ferritin (Fer), B symptoms, white blood cell count (WBC), red blood cell count (RBC), hemoglobin (HGB), neutrophil count (NE), cell-of-origin (COO), presence or absence of hepatosplenomegaly, therapeutic regimens, and immunological markers (MYC, BCL-2, BCL-6, and Ki-67). Cases were staged according to the Ann Arbor staging system, and IPI scores were also evaluated.

### Pathological Studies

All cases were diagnosed according to the WHO criteria. The exclusion criteria were as follows: 1) primary central nervous system and testicular lymphoma, 2) primary mediastinal large B-cell lymphoma, 3) transformed large B-cell lymphoma, 4) infected with human immunodeficiency virus, and 5) combined with other malignant tumors.

Biopsy samples were fixed in formalin, embedded in paraffin, sliced, and stained with hematoxylin and eosin for morphological analysis. Morphologically, all cases of DLBCL were categorized into three types: centroblastic, immunoblastic, and anaplastic variants. The centroblastic variant was shown to have large-sized nuclei with two to four small nucleoli closed to the nuclear membrane. Large, bizarre, and irregular cells were observed among tumor cells. The immunoblastic subtype was characterized by a rich cytoplasm with nucleolus laid in the center, and a local cytoplasm-like differentiation could be observed. Large to very large cells with bizarre pleomorphic nuclei were observed in the anaplastic variant, which may resemble the neoplastic cells of anaplastic large cell lymphoma.

Expression of biomarkers CD5, MYC, BCL-2, BCL-6, and Ki-67 was assessed using respective antibodies. All the histopathology sections were confirmed by at least two expert pathologists. Cutoff points for MYC, BCL-2, and BCL-6 proteins were designated as 40%, 50%, and 50% staining positive on lymphoma cells. It could be considered as CD5-positive when large tumor B cells express CD5 (>20%) as well as pan B markers (CD20, Pax5, CD19, etc.). GCB or non-GCB phenotypes were determined by the Hans algorithm (26).

### Follow-Up and Endpoints

Follow-up was conducted by consulting inpatient medical records and making phone calls. We followed up all the patients until July 12, 2021, or the death of patients. Overall survival (OS) was calculated as the interval between the time of diagnosis and death from any cause or the last follow-up. The survival status of all patients was confirmed with death records or a telephone call to next of kin (if patient died during the follow-up) or to the patients themselves.

### Statistical Analysis

Data were presented as numbers (percentages) for categorical variables and median (interquartile range, IQR) for all continuous variables. Outliers were verified by the hospital medical record system. Differences in clinicopathologic factors were analyzed by using the Mann–Whitney *U* test and *χ* ^2^ test. Continuous variables were transformed into categorical variables by MaxStat analysis (titled as maximally selected rank statistics).

The potential impact of interrelationships among independent variables was evaluated by collinearity analysis. The degree of collinearity can readily be assessed by variance inflation factor (VIF) statistic (27). To evaluate the distance between variables and the presence of clusters, an r-type clustering analysis was utilized. The Cox proportional hazard model was used to analyze the univariate association between prognostic factors and OS. All variables with *P <*0.05 in univariable analysis were kept in the multivariable analysis by using forward selection for the best predictor set and the Akaike information criteria (AIC) were used to evaluate the model. The model was internally validated using a bootstrap resampling procedure (500 iterations) with a relatively corrected Harrell’s C-statistics (C-index), and the calibration curve was calculated according to the regression results. Brier score is another score function that measures the accuracy of probabilistic prediction. The nomogram was constructed on the grounds of the Cox model parameter estimates in the derivation cohort. The visual format of the nomogram reflects a statistical prediction that can determine how many points are attributed for each variable value, and the relative importance of predictors can be judged by the length of each line within the nomogram (25). All statistical tests were two-sided and statistical significance was set at *P <*0.05. The statistical analyses were performed by SPSS statistics for Windows, Version 19.0 (Armonk, NY: IBM Corp.) and R software (version 4.0.3; http://www.Rproject.org).

## Results

### Clinical Characteristics

A total of 195 CD5+ DLBCL cases were selected from 1,864 DLBCL patients. The derivation cohort consisted of 131 cases from two centers and the external validation cohort consisted of 64 patients from seven centers. At the end of follow-up, a total of 81 (41.54%) deaths occurred. The median follow-up was 37.8 months [95% CI (35.9–39.7)] and the median survival time was 55.2 months [95% CI (39.0–71.4)]. The 5-year OS of patients was 42.1%. Median age at diagnosis was 61 years (range: 24–90), of whom 104 (53.33%) were older than 60 years and 100 (51.28%) were males. Ann Arbor stage III/IV accounted for 57.95%. The characteristics of patients in the two cohorts are detailed in **Table 1**.

**Table 1:** Characteristics of CD5+ DLBCL patients in the two cohorts.

Variables	Derivation cohort	Validation cohort	*P*	*χ* ^2^/*Z*
	*n* = 131	*n* = 64		
Age^a^	65 (53–70)	57 (56–70)	0.327	−0.98
Alb^a^	36.1 (30.2–40.7)	35 (34.7–39.3)	0.604	−0.519
NE^a^	3.7 (2.3–4.5)	2.9 (2.8–3.7)	0.743	−0.329
RBC^a^	4.2 (4.0–4.5)	3.9 (3.3–4.1)	0.100	−1.644
LYC^a^	1.4 (1.0–1.5)	1.7 (1.2–2.7)	0.924	−0.095
LDH^a^	217 (162–310)	288 (218–689)	0.492	−0.687
Fer^a^	188.5 (124.1–294.0)	137.9 (129.5–274.0)	0.394	−0.853
β2-MG^a^	2.2 (2.0–3.9)	3.7 (2.3–7.3)	<0.001	−4.301
Gender: male^b^	70 (53.4%)	30 (46.9%)	0.389	0.741
ECOG PS (2–4)^b^	27 (20.6%)	21 (32.8%)	0.063	3.450
Stage III/IV^b^	70 (53.4%)	46 (71.9%)	0.014	6.066
BM involvement^b^	9 (6.9%)	10 (15.6%)	0.001	20.185
CNS involvement^b^	16 (12.2%)	16 (25.0%)	0.024	5.124
B symptoms^b^	37 (28.2%)	14 (22.9%)	0.541	0.373
Bulky^b^	3 (2.3%)	4 (6.6%)	0.139	2.194
Non-GCB^b^	74 (56.5%)	45 (70.3%)	0.063	3.455
Ki-67 ≥0.9^b^	24 (18.3%)	10 (15.6%)	0.641	0.217
MYC^b^	81 (61.8%)	27 (42.2%)	0.001	13.389
BCL-2+^b^	88 (67.2%)	41 (64.1%)	0.666	0.186
BCL-6+^b^	85 (64.9%)	30 (46.9%)	0.016	5.764

a Continuous variables were presented in median and interquartile range.

b Categorical variables were presented in numbers and percentages.

Alb, albumin; NE, neutrophil count; RBC, red blood cell count; LYC, lymphocyte count; LDH, lactate dehydrogenase; Fer, ferritin; β2-MG, β2-microglobulin; BM involvement, bone marrow involvement; CNS involvement, central nervous system involvement.

### Survival Analysis of the Whole Cohort

**Figure 1:**
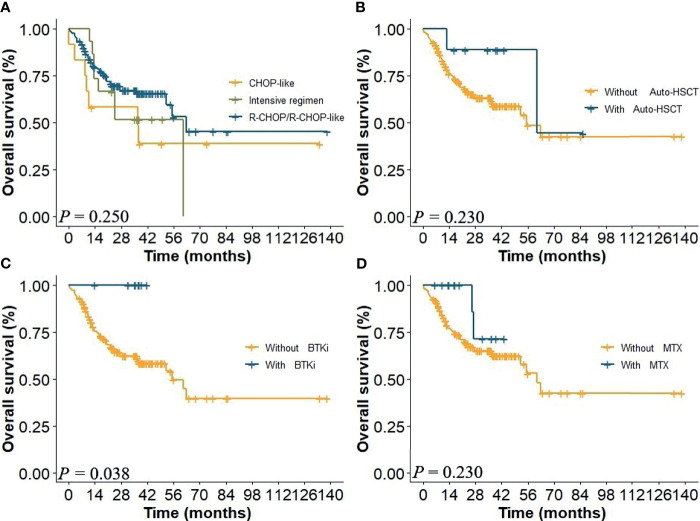
**(A)** Overall survival (OS) of patients in the whole cohort of different therapeutic regimens and with or without **(B)** auto-HSCT, **(C)** BTKi, and **(D)** MTX.

In this study, patients received regimens of CHOP-like (*n* = 12), R-CHOP/R-CHOP-like (*n* = 122), and R-based intensive regimens (*n* = 15). Nine patients received BTK inhibitor (BTKi), nine patients received methotrexate (MTX), and 14 patients received autologous hematopoietic stem cell transplantation (auto-HSCT). Kaplan–Meier analysis indicated that there was no significant difference in therapeutic regimens on the prognosis of CD5+ DLBCL in global comparison (*P* = 0.250, **Figure 1A**). Further analysis on specific regimens showed that patients in BTKi regimen showed a better survival than those treated without BTKi (*P* = 0.038, **Figure 1C**). However, there was no statistical difference in the regimens of auto-HSCT and R-CHOP with MTX (**Figures 1B, D**).

**Figure 2:**
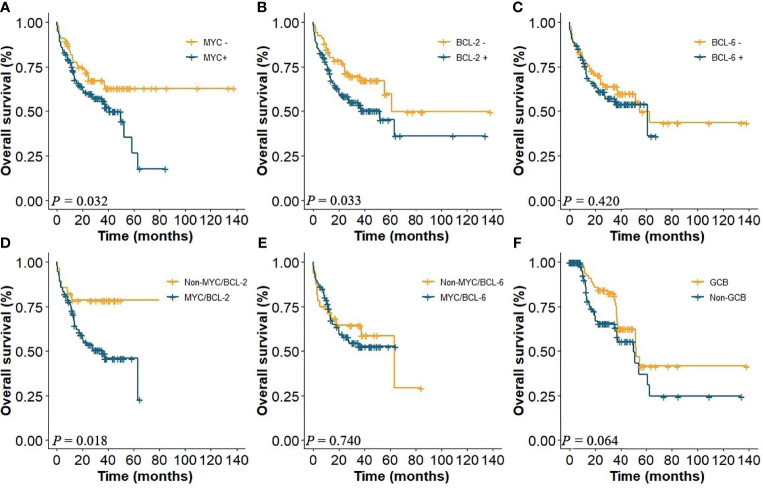
**(A)** OS of CD5+ DLBCL patients with the presence or absence of MYC, **(B)** with the presence or absence of BCL-2, and **(C)** with the presence or absence of BCL-6. **(D)** Coexpression of MYC/BCL-2; **(E)** coexpression of MYC/BCL-6; **(F)** COO subtypes.

Using cutoff values of 40%, 50%, and 50% positive tumor cells for MYC, BCL-2, and BCL-6, respectively, 108 (55.4%) were positive for MYC, 129 (66.2%) cases were positive for BCL-2, and 115 (58.9%) cases were positive for BCL-6. Thirty-four (17.4%) patients were with high Ki-67 score (≥0.9) and 119 (61.0%) were non-GCB. MYC and BCL-2 coexpression in CD5+ DLBCL had a significant adverse impact on patient survival (**Figure 2D**), while MYC and BCL-6 coexpression was not a significant factor for OS (**Figure 2E**). The 5-year OS of CD5+ DLBCL patients with MYC/BCL-2 coexpression was 23% (*P* < 0.018, **Figure 2D**). When assessed separately, patients with BCL-2+ or MYC+ had significantly inferior OS (**Figures 2A, B**) compared with patients with BCL-2− or MYC−, respectively. However, patients with BCL-6+ was not an adverse factor for OS (**Figure 2C**). Similarly, COO subtypes and Ki-67 were not predictive factors for CD5+ DLBCL survival (**Figure 2F**, *P* = 0.064; data not shown for Ki-67, *P* = 0.118).

### The Cutoff Points for Continuous Variables Based on MaxStat

**Figure 3:**
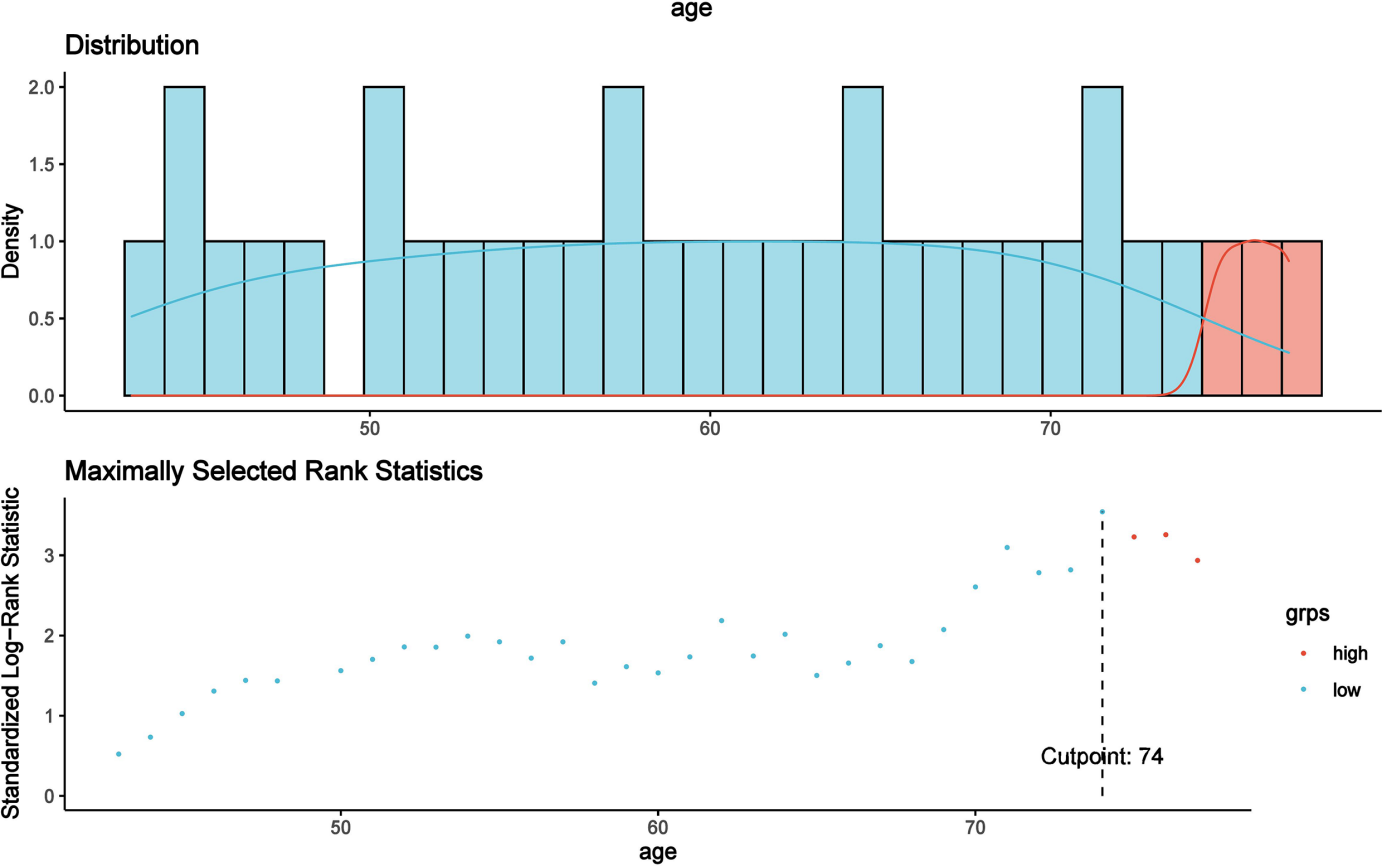
The optimal cutoff point for age was found to be 74 years using the MaxStat method, which distinguished two prognostic groups most effectively (*P* < 0.0001).

According to the MaxStat method, 74 years, 39.5 g/L, 6 × 10^9^/L, 3.87 × 10^9^/L, 3.62 × 10^12^/L, 106 g/L, 139 × 10^9^/L, 483 U/L, and 0.9 × 10^9^/L were the optimal cutoff points for age, Alb, WBC, NE, RBC, HGB, PLT, LDH, and LYC that distinguished the two groups most effectively (*P* < 0.05, **Figure 3**).

### Univariable and Multivariable Analyses

The results suggested that there were no interrelationships among independent variables (VIF < 7, *R* = 0.695). After cluster analysis, the original variables were divided into four categories. Age, RBC, IPI, MYC, and other clinical characteristics were statistically significant (*P* < 0.05). Univariable analysis exhibited that IPI, RBC, Alb, CNS involvement, BM involvement, and hepatosplenomegaly were strong prognostic predictors (*P* < 0.001). Following the model iterations in multivariable analysis, the final prognostic index consisted of six factors, as shown in **Table 2**. IPI, NE, MYC, hepatosplenomegaly, and age proved to be adverse factors for the survival of CD5+ patients. Nevertheless, CNS involvement in the current multivariable model was observed to be not predictive [*P* = 0.129, HR = 1.808, 95% CI (0.841–3.885)].

**Table 2:** Multivariable analysis of OS in the derivation cohort.

Variables	HR	95% CI	*P*
IPI			
LR + LIR			
HIR + HR	3.298	1.753–6.204	<0.001
RBC			
<3.62			
≥3.62	0.291	0.143–0.590	0.001
NE			
<3.87			
≥3.87	2.949	1.535–5.665	0.001
MYC			
Negative			
Positive	3.597	1.245–10.392	0.018
Hepatosplenomegaly			
Absence			
Presence	2.608	1.110–6.127	0.028
Age			
<74			
≥74	2.045	1.042–4.014	0.038

IPI, International Prognostic Index; RBC, red blood cell count; NE, neutrophil count.

### Development of the Nomogram and Comparison to IPI

**Figure 4:**
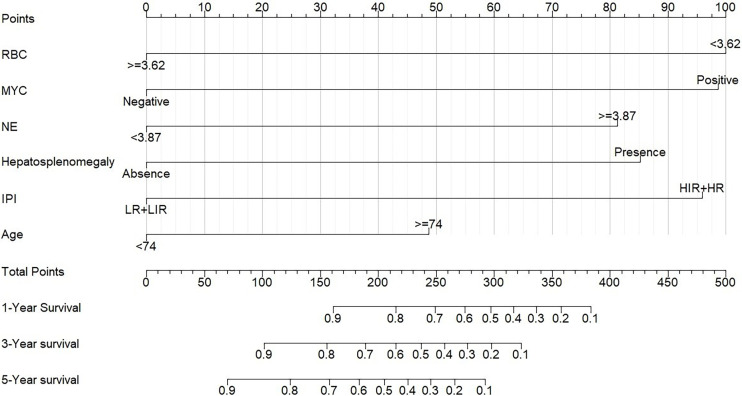
Nomogram for patients with CD5+ DLBCL. To use the nomogram, the specific points of individual patients are located on each variable axis. Black lines are drawn upward to determine the points received by each variable.

**Figure 5:**
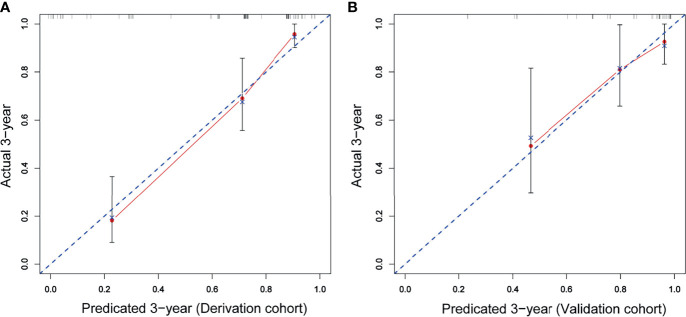
The red solid line represents the performance of the nomogram, and the higher the fitting degree with the diagonal dotted line, the better the prediction effect. **(A)** Derivation cohort; **(B)** validation cohort.

**Figure 6:**
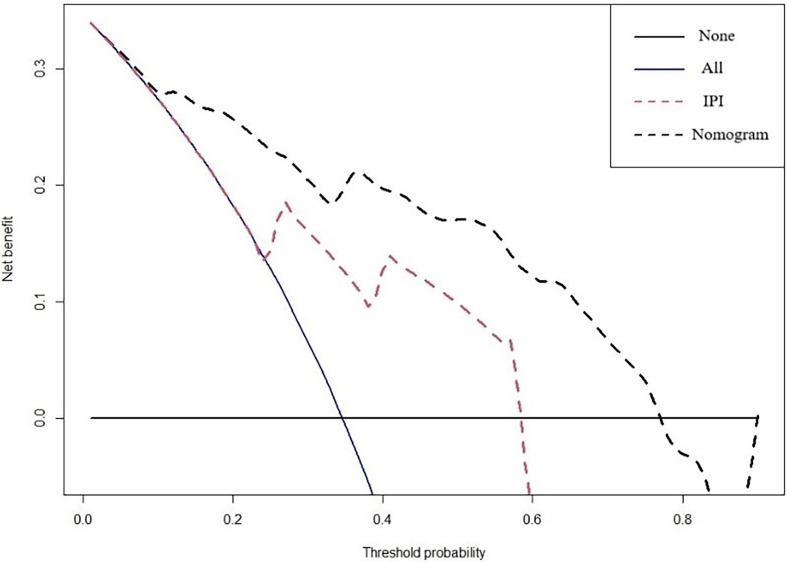
DCA curves to evaluate the clinical utility of different decision strategies. The red line represents the IPI system, and the black line represents the nomogram for CD5+ DLBCL.

A prognostic nomogram was developed to predict 1-, 3-, and 5-year OS of CD5+ DLBCL patients based on multivariable analysis (**Figure 4**). The C-index (0.809) and the Brier score (0.151) were calculated between the predicted outcome and the real outcome of the model for internal validation. We further validated this nomogram externally and computed the C-index and the Brier score in an independent validation cohort of 64 patients (C-index = 0.770 and Brier score = 0.241). The calibration curves were close to the ideal curves, suggesting that the predicted result and the actual outcome had a good consistency (**Figure 5**). In this study, all cases had complete data for all the variables required to calculate the IPI score. DCA was used to calculate the clinical net benefit of each model compared with all or none strategies, and the result demonstrated that the nomogram showed the best net benefit for 5-year OS (**Figure 6**). Taken together, these results indicated that compared with IPI, the nomogram was more appropriate for predicting the survival of CD5+ DLBCL patients.

## Discussion

Due to the heterogeneity of DLBCL, identification of subtypes and precise prognostic evaluation are needed for individualized treatment (28, 29). CD5+ DLBCL is a specific immune subtype of DLBCL with an aggressive clinical course. In this study, we retrospectively analyzed the clinicopathologic characteristics and developed the nomogram to predict the prognosis of CD5+ DLBCL patients for clinical individualized treatment guidance.

CD5+ DLBCL is prone to elderly onset, advanced stage at diagnosis, and elevated LDH level, and the 5-year OS is less than 40% in the era of rituximab-based immunochemotherapy (30, 31). In addition, patients of CD5+ DLBCL were with frequent CNS relapses and genetic abnormality (17, 18, 20, 32). Miyazaki et al. demonstrated that DA-EPOCH-R/HD-MTX could be a first-line therapy option for stage II–IV CD5+ DLBCL, improving 2-year OS to 89% (33). In this study, we found that 53.33% patients were older than 60 years and 59.49% patients were with advanced stage (III + IV) which were consistent with previous studies. Totally, the 5-year OS of CD5+ DLBCL was 42.1% in our study, and there was no statistical difference among the CHOP-like regimen, R-CHOP regimen, and R-based intensive regimen. In addition, in this retrospective study, positive effects of auto-HSCT and MTX on the survival of patients were not observed. However, nine patients with BTKi regimen demonstrated superior survival than those without. The intrinsic characteristics and individualized treatment need to be further investigated.

DLBCL is highly heterogeneous in pathological features. Hans et al. proved that compared with GCB, non-GCB patients had poorer outcomes with a 5-year OS of only 34% (26). In this study, the difference between GCB and non-GCB subtypes was not statistically significant. In addition, we evaluated the prognosis values of pathological features, such as MYC, BCL-2, BCL-6, Ki-67, coexpression of MYC/BCL-2, and coexpression of MYC/BCL-6 on CD5+ DLBCL. The results confirmed that BCL-6 and Ki-67 were not associated with survival, while BCL-2 and MYC were independent adverse predictors. Further investigation indicated that the status of MYC/BCL-2 could distinguish the survival of CD5+ DLBCL, whereas MYC/BCL-6 coexpression could not. So, our data suggested that the poor prognosis of CD5+ DLBCL might be independent of COO, BCL-6, and coexpression of MYC/BCL-6.

To explore the prognostic factors of CD5+ DLBCL, alternative clinicopathological variables were included and collinearity analysis was conducted before univariable analysis. Subsequently, we developed a specific nomogram based on the multivariable Cox model, which consisted of six variables: age, IPI, RBC, MYC, hepatosplenomegaly, and NE. In order to assess the accuracy and discrimination of the nomogram, it has been externally validated to predict survival in seven medical centers. The C‐index and the Brier score of the nomogram were 0.809 and 0.151 in internal validation and 0.770 and 0.241 in external validation. In contrast to the IPI, the DCA curve showed that the nomogram was better in predicting the prognosis of CD5+ DLBCL patients.

Due to the inherent flaws of the retrospective design, the lack of genetic measurements, and the limitation of sample size, further prospective multicenter study is urgently needed to validate the model. In conclusion, we retrospectively analyzed the clinicopathological characteristics of CD5+ DLBCL patients from a multicenter in China and developed the novel nomogram, providing a valuable tool for prognosis prediction.

## Data Availability Statement

The raw data supporting the conclusions of this article will be made available by the authors, without undue reservation.

## Ethics Statement

Study approval was obtained from the independent ethics committees of each participating center in HHLWG. The ethics committee waived the requirement of written informed consent for participation.

## Author Contributions

Conception and design: WS. Drafting of the manuscript: ZS and LW. Analysis and interpretation of data: ZS. Acquisition of data: BZ, TL, DL, CH, YX, YW, BL, QL, HZ, WG, FW, CW, YS, JY, TZ, and YM. Supervision: WS and SH. All authors contributed to the article and approved the submitted version.

## Funding

This study was funded by the Natural Science Foundation of Jiangsu Province, Grant/Award Number: BK20171181; Jiangsu Key Research and Development Project of Social Development, Grant/Award Number: BE2019638; and Young Medical Talents of Jiangsu Science and Education Health Project, Grant/Award Number: QNRC2016791.

## Conflict of Interest

The authors declare that the research was conducted in the absence of any commercial or financial relationships that could be construed as a potential conflict of interest.

## Publisher’s Note

All claims expressed in this article are solely those of the authors and do not necessarily represent those of their affiliated organizations, or those of the publisher, the editors and the reviewers. Any product that may be evaluated in this article, or claim that may be made by its manufacturer, is not guaranteed or endorsed by the publisher.
